# Fibrodysplasia ossificans progressiva: genetic and clinical characterization in a cohort of Polish patients and review of potential therapies

**DOI:** 10.1007/s13353-025-00966-4

**Published:** 2025-04-12

**Authors:** Anna Szoszkiewicz, Małgorzata Szczepanek, Ewelina Bukowska-Olech, Anna Sowińska-Seidler, Magdalena Socha, Aleksander Jamsheer

**Affiliations:** 1 Poznan University of Medical Sciences, Department of Medical Genetics, Rokietnicka 8, Poznan, Poland; 2 Poznan University of Medical Sciences, Doctoral School, Department of Medical Genetics, Poznan, Poland; 3 University of Rzeszow, Faculty of Medicine, Rzeszów, Poland; 4 2nd Department of Pediatrics, Endocrinology and Diabetology, Clinical Provincial Hospital No. 2, Rzeszów, Poland; 5 Adam Mickiewicz University, Institute of Molecular Biology and Biotechnology, Poznan, Poland; 6 Diagnostyka GENESIS, Dąbrowskiego 77A, 60 - 529 Poznan, Poland; 7 Poznan University of Medical Sciences, Department of Laboratory Diagnostics, Poznan, Poland

**Keywords:** Heterotopic ossification, Activin A type I receptor gene (*ACVR1*), Genotype–phenotype correlations, Bone morphogenetic protein (BMP), Treatment strategies

## Abstract

Fibrodysplasia ossificans progressiva (FOP; OMIM #135100) is a rare genetic disorder characterized by congenital malformation of the great toes and progressive heterotopic ossification of soft tissues. To date, the disease has been linked to 15 pathogenic variants in the *ACVR1* gene, which encodes a type I receptor for bone morphogenetic proteins. Most patients with FOP carry a recurrent single-nucleotide substitution (c.617G>A; p.Arg206His) in the *ACVR1* gene. The genotype–phenotype correlations for atypical pathogenic variants of *ACVR1* are poorly understood. In this study, we report the largest population of Polish patients affected by FOP and analyze their phenotypes and genotypes. We screened the whole *ACVR1* coding sequence of 16 patients affected by FOP to confirm the presence of pathogenic variants. Thirteen individuals carried the classic pathogenic variant (p.Arg206His) and had a classic or FOP-plus phenotype. In agreement with the findings of previous studies, one patient with a p.Gly356Asp pathogenic variant had a variant FOP phenotype. We point to an unusual phenomenon in two patients who carried atypical pathogenic variants (p.Gly356Asp and p.Arg258Ser) and displayed a classic FOP phenotype. Our study extends the understanding of FOP’s genotype–phenotype correlation, suggesting that classic FOP phenotypes are associated with non-classic pathogenic variants. We also summarize the recent advances in drug development for this condition. Therefore, the study may be valuable for clinicians consulting patients with FOP.

**Supplementary Information:**

The online version contains supplementary material available at 10.1007/s13353-025-00966-4.

## Introduction

Fibrodysplasia ossificans progressiva (FOP; OMIM #135100) is a rare, autosomal dominant disorder characterized by heterotopic bone formation (heterotopic ossification, HO) in the connective tissue and skeletal muscles. HO is preceded by inflammatory swelling of soft tissues, known as flare-ups, and leads to progressive ankylosis of joints, resulting in restricted movement. Although flare-ups occur intermittently, the resulting disability accumulates over time. Consequently, most individuals with FOP require wheelchairs by their third decade of life. The prevalence of FOP is estimated to be 0.88 in 1 million people, regardless of race, geographic predisposition, and gender (Pignolo et al. 2013, 2021). The classic clinical features of the disease are progressive HO and a congenital, bilateral malformation of the great toes, i.e., hallux valgus, deformity of the first metatarsal, and monophalangism. Other symptoms include a broad, short femoral neck, hearing loss, proximal medial tibial osteochondromas, cervical vertebrae fusions, thumb malformations, dental abnormalities, cardiopulmonary complications, and neurologic dysfunction (Kaplan et al. 2009). FOP is classified into three groups based on clinical criteria: the classic phenotype, which has two major clinical features, i.e., malformed great toes and progressive HO, and the other common FOP symptoms in > 50% of patients; the FOP-plus phenotype, in which patients have the classic FOP symptoms and atypical features; and the FOP variant with major variations in one or both of the classic defining features of FOP (Kaplan et al. 2009). The average life expectancy of patients with FOP is 56 years, and death is often due to cardiorespiratory complications of thoracic insufficiency syndrome (Pignolo et al. 2021). Treatment, including glucocorticoids and nonsteroidal anti-inflammatory drugs, focuses on symptomatic relief (Smilde et al. 2022).

**Fig. 1:**
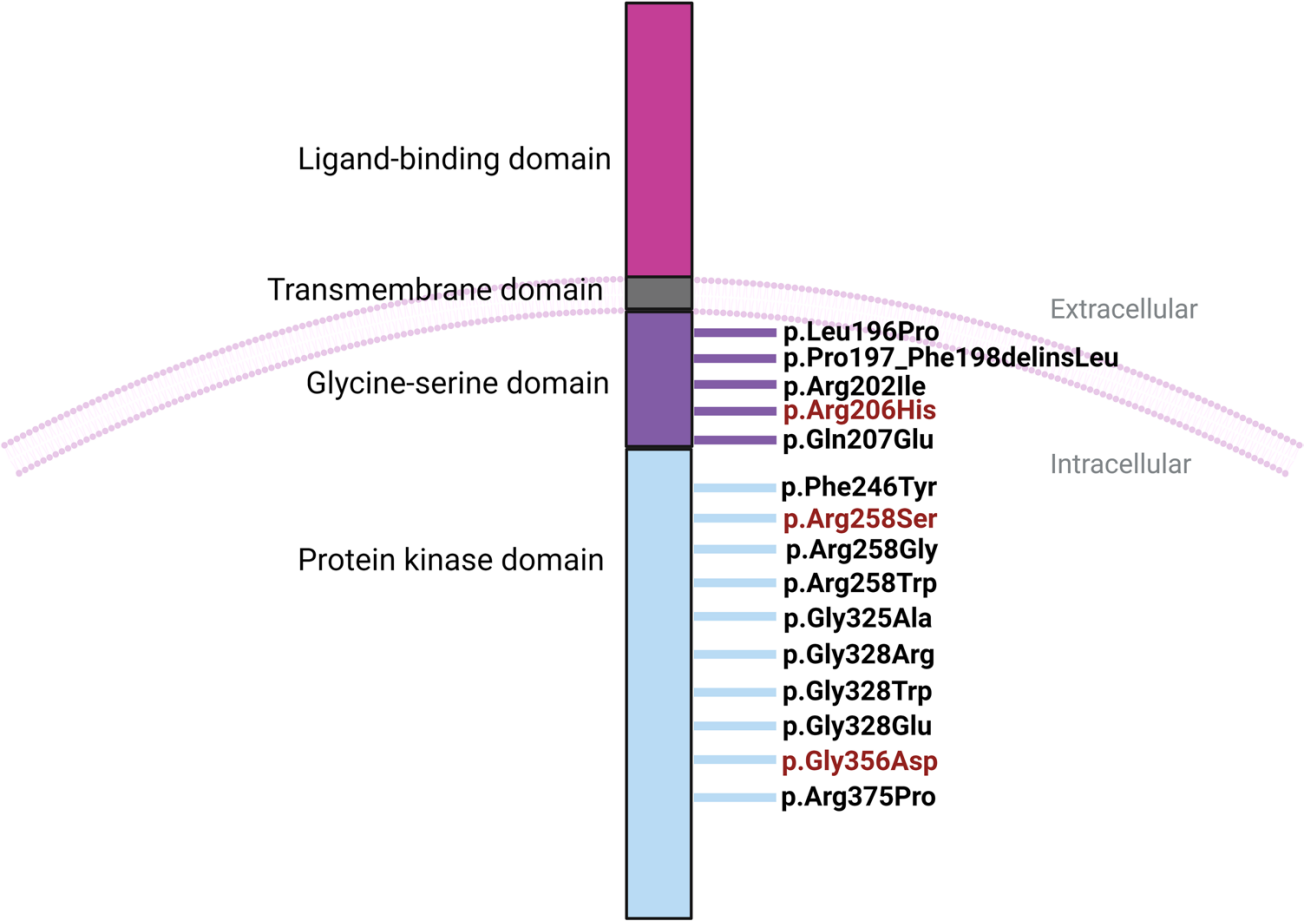
Schematic representation of human ACVR1 protein structure, showing the locations of all pathogenic variants associated with FOP. The pathogenic variants observed in the patients of our cohort are marked with red color. Created with Biorender.com

**Fig. 2:**
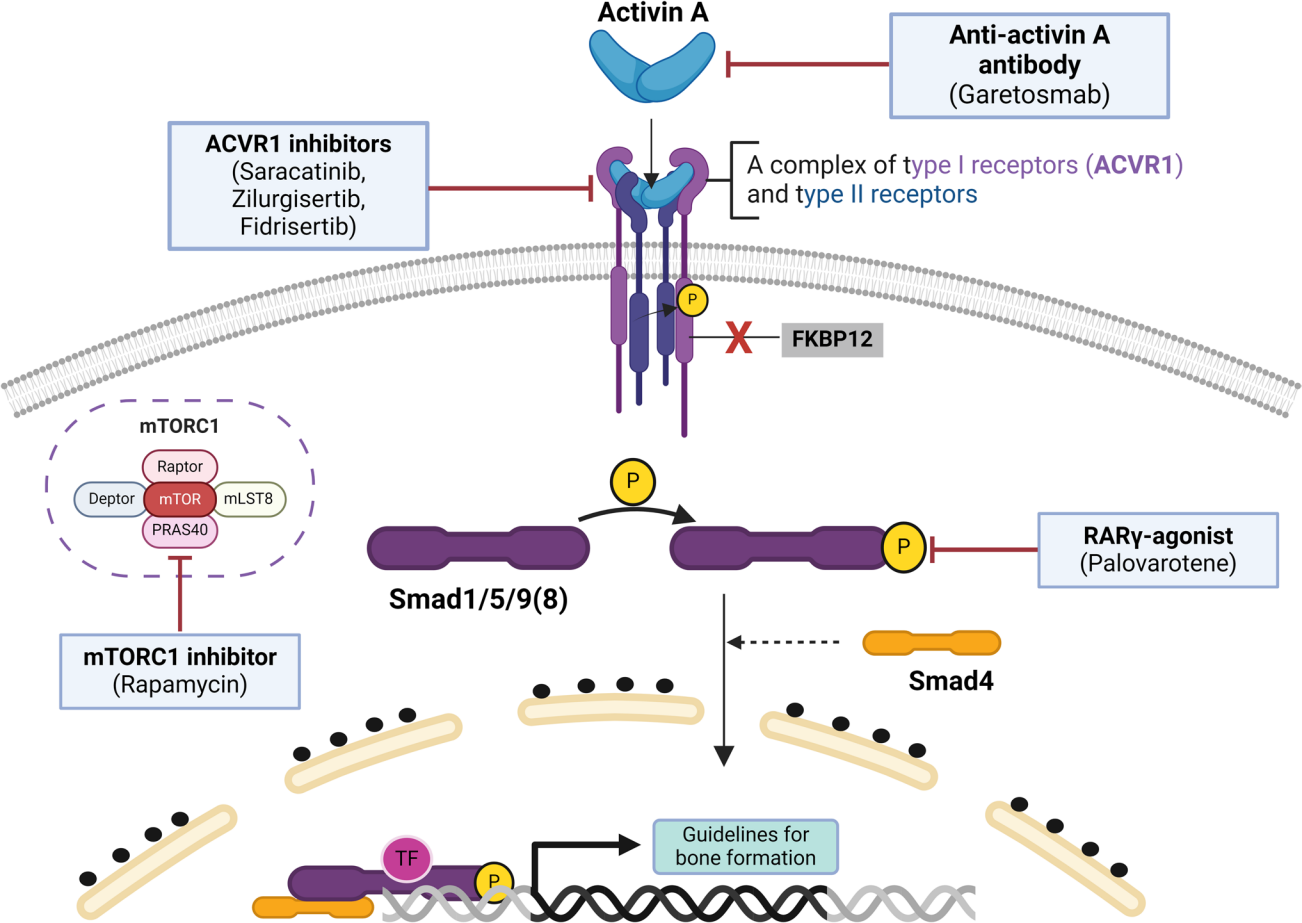
Dysregulated BMP signaling pathway in FOP and potential treatment options under clinical trials. Created with Biorender.com

FOP results from pathogenic variants in the *ACVR1* gene. This gene encodes activin receptor type- 1 (ACVR1), a receptor of the bone morphogenetic protein (BMP) signaling pathway, which is crucial for the development of the skeletal system. ACVR1 belongs to the transforming growth factor-beta receptor (TGFBR1) family and forms a tetrameric complex at the cell membrane in conjunction with type II receptors. Both types of receptors contain three common domains: a ligand-binding domain, a transmembrane domain, and a serine/threonine kinase domain. Additionally, type I receptors have the glycine-serine (GS) domain (Fig. 1), the site for transferring phosphorylation from a type II receptor to a type I receptor (Wrana et al. 1994). In this process, the 12 kDa FK506-binding protein (FKBP12) represses the kinase activity of type I receptors by binding to the unphosphorylated GS domain. The pathogenic variant in the *ACVR1* gene induces hyperactivity of the ACVR1 receptor in response to ligands and, in turn, triggers the phosphorylation of the GS domain, leading to structural alterations within the domain. This alteration results in the detachment of the previously bound FKBP1 A from the GS domain. The phosphorylated ACVR1 type I receptor activates SMAD1/5/9(8) phosphorylation, which binds to SMAD4. The complex translocates to the nucleus, where it activates transcription factors and the expression of BMP target genes (Fig. 2). Consequently, affected patients experience progressive HO of soft connective tissues (Song et al. 2010).

**Fig. 3:**
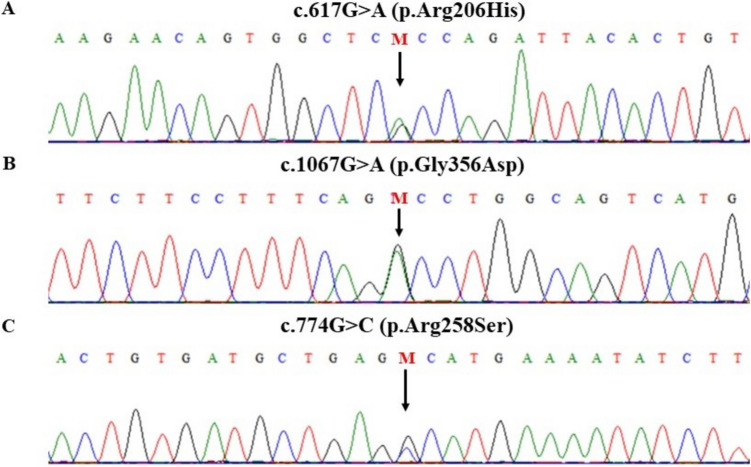
DNA sequencing electropherograms of the three heterozygous pathogenic variants found in our cohort: the c.617G>A pathogenic variant (**A**), c.1067G>A pathogenic variant (**B**), and c.774G>C pathogenic variant (**C**)

The most common pathogenic variant found in approximately 97% of FOP patients is c.617G>A, which transforms arginine to histidine at position 206 (p.Arg206His) (Shore et al. 2006; Hatsell et al. 2015). The p.Arg206His pathogenic variant results in GS domain disruption and hyperactivation of the BMP signaling pathway. In addition to the classical pathogenic variant, FOP has been linked to 14 other pathogenic variants in *ACVR1*, i.e., c.590_592delCTT (p.Pro197_Phe198delinsLeu), c.619C>G (p.Gln207Glu), c.983G>A (p.Gly328Glu), c.982G>A (p.Gly328Arg), c.982G>T (p.Gly328Trp), c.1067G>A (p.Gly356Asp), c.1124G>C (p.Arg375Pro), c.605G>T (p.Arg202Ile), c.774G>C (p.Arg258Ser), c.587T>C (p.Leu196Pro), c.974G>C (p.Gly325 Ala), c.772G>A (p.Arg258Gly), c.737T>C (p.Phe246Tyr), and c.772 A>T (p.Arg258Trp) (Kaplan et al. 2009, 2022; Bocciardi et al. 2009; Gregson et al. 2011; Petrie et al. 2009; Rafati et al. 2016; Whyte et al. 2012; Furuya et al. 2008; Ratbi et al. 2010; Carvalho et al. 2010). All the pathogenic variants associated with FOP are located either in the GS domain or the protein kinase domain of ACVR1 (Fig. 1). The classical pathogenic variant was reported in all FOP phenotypes, while atypical pathogenic variants were mainly associated with FOP-plus and FOP variant phenotypes (Huning and Gillessen-Kaesbach 2014). FOP usually occurs due to de novo pathogenic variants, while less frequently, it is inherited from an affected parent in an autosomal dominant manner (Shore et al. 2006) (Fig. 3).

To date, a few cases of FOP have been reported in Poland (Dulski and Sławek 2020; Dąbrowska et al. 2019; Puszczewicz et al. 2007). Moreover, only four publications comprise a large group of patients with this condition (Kaplan et al. 2009; Bocciardi et al. 2009; Carvalho et al. 2010; Zhang et al. 2013). This is the first study of such a large cohort of FOP patients in Poland and the fifth globally. In this paper, we conduct a detailed clinical evaluation and genetic analysis of *ACVR1* on 16 Polish patients affected by FOP. Our study aims to compare the genotype–phenotype correlations between the Polish population and other populations. We evaluate the prevalence of known *ACVR1* pathogenic variants and search for new ones within our cohort. Our paper also reviews recent advances in FOP drug development.

## Materials and methods

The Ethics Committee of Poznan University of Medical Sciences approved this study. We obtained written informed consent from 16 patients diagnosed with FOP before collecting peripheral blood or buccal swab samples. We extracted genomic DNA (gDNA) from venous blood using the MagCore® HF16 Automated Nucleic Acid Extractor (RBC Bioscience Corp.) or the Nucleo-Spin Tissue Kit (Machery-Nagel) for buccal swabs. gDNA was quantified using a Nanodrop 2000 (Thermo Scientific). Genetic testing was performed by amplifying nine coding exons of the *ACVR1* gene (RefSeq NM_001111067.4) by polymerase chain reaction (PCR) in a total volume of 10 μl containing 5 μl of FailSafe™ PCR 2X PreMix J (Lucigen Epicenter), 2.9 μl of PCR-grade water, 0.5 μl of primers (10 μmol/l each), 0.1 μl of Taq DNA polymerase (GenScript), and 1 μl of gDNA. The PCR conditions were described previously (Bukowska-Olech et al. 2020). We checked the amplified DNA by standard agarose gel electrophoresis and purified it following standard protocols. The primers were designed using the Primer3 tool v. 0.4.0 (Table S1). Next, PCR products were sequenced using dye-terminator chemistry (kit v.3, ABI 3130xl) according to the provided protocol and run on a PRISM 3700 DNA Analyzer (Applied Biosystems). We analyzed the sequence data using the BioEdit tool. The reaction conditions are available upon request.

## Results

### Molecular results

Our study examined 16 Polish patients clinically diagnosed with FOP. We conducted a screening of the entire coding region of the *ACVR1* gene and identified three pathogenic variants (c.617G >A, c.1067G>A, and c.774G>C). No additional variants were detected. We summarized the genotype and phenotype of all patients in Table 1. Most of our cohort, representing 13 (81.25%) individuals, had the classic missense heterozygous pathogenic variant c.617G> A (p.Arg206His) in the *ACVR1* gene (NM_001111067.4). Two patients (12.5%) carried a heterozygous c.1067G> A (p.Gly356Asp) pathogenic variant, and one patient (6.25%) had a heterozygous c.774G>C (p.Arg258Ser) pathogenic variant in the *ACVR1* gene. We confirmed the de novo origin of the pathogenic variants in all patients by performing Sanger sequencing on the patients and their parents. In line with the literature, we selected specific phenotypic features for analysis (Kaplan et al. 2009). Phenotypic data were unavailable for P6 due to his death. Our cohort predominantly included female patients. The mean age of ossification onset was 3.7 years. The disease manifested severely in 63% of patients. According to clinical classification schemes, we categorized patients into three phenotypic groups, which we described below.

**Table 1:** Comparison of the genotype and phenotype among 16 FOP patients

	P1	P2	P3	P4	P5	P6	P7	P8	P9	P10	P11	P12	P13	P14	P15	P16
Clinical features																
Pathogenic variant	c.617G>A, p.Arg206His de novo													c.1067G>A p.Gly356Asp de novo		c.774G>C p.Arg258Ser de novo
Gender	F	F	F	M	F	M	F	F	M	F	F	F	M	F	M	F
Age ossification onset	3 ys	10 mo	3 ys	3 ys	11 mo	ND	3 ys	1 y	6 ys	11 ys	2.5 ys	4 ys	3 ys	9 mo	9 mo	13 ys
Disease progression	Mod	Sev	Sev	Sev	Mild	ND	Sev	Mod	Sev	Sev	Mod	Sev	Sev	Sev	Mod	Sev
Classic FOP features																
Progressive HO	+	+	+	+	+	ND	+	+	+	+	+	+	+	+	+	+
Great toe MFs	+	+	+	+	+	ND	+	+	+	+	+	+	+	+	+	+
**Common FOP features**																
Thumb MFs	** + **	** + **	** + **	** + **	** + **	ND	** + **	** + **	** + **	** + **	** + **	** + **	** + **	** + **	** + **	** + **
Short broad femoral neck	** + **	** + **	** + **	** + **	** + **	ND	** + **	** + **	** + **	** + **	** + **	** + **	** + **	** + **	** + **	** + **
Tibial osteochondromas	** + **	** + **	** + **	** + **	** + **	ND	** + **	** + **	** + **	** + **	** + **	** + **	** + **	** + **	** + **	** + **
Cervical vertebral MFs	** + **	** + **	** + **	** + **	** + **	ND	** + **	** + **	** + **	** + **	** + **	** + **	** + **	** + **	** + **	** + **
Conductive hearing impairment	** − **	** + **	** + **	** + **	** − **	ND	** + **	** + **	** + **	** + **	** − **	** − **	** + **	** + **	** + **	** + **
**Less common FOP features**																
Neurological dysfunction	** + **	** − **	** + **	** − **	** − **	ND	** − **	** − **	** − **	** − **	** − **	** − **	** − **	** − **	** − **	** − **
Cardiopulmonary complications	** − **	** − **	** + **	** + **	** − **	−	** + **	** + **	** + **	** + **	** + **	** + **	** + **	** − **	** − **	** + **
**Atypical FOP features**																
Reduction deficits of digits	** − **	** − **	** − **	** − **	−	ND	−	** − **	−	−	** − **	** − **	** − **	** − **	+	−
Synovial chondrosarcoma of hips	** − **	** − **	** − **	** − **	+	ND	−	** − **	−	−	** − **	** − **	** − **	** − **	−	−
Sparse, thin scalp hair	** + **	** − **	** + **	** + **	−	ND	−	** − **	+	−	** − **	** − **	** − **	** − **	−	−

*P* patient, *MFs* malformations, *Mod* moderate, *Sev* severe, *HO* heterotopic ossification, *ND* no data, *mo* months, *y(s)* year(s)

### Clinical phenotypes

#### Patients with classic FOP

**Fig. 4:**
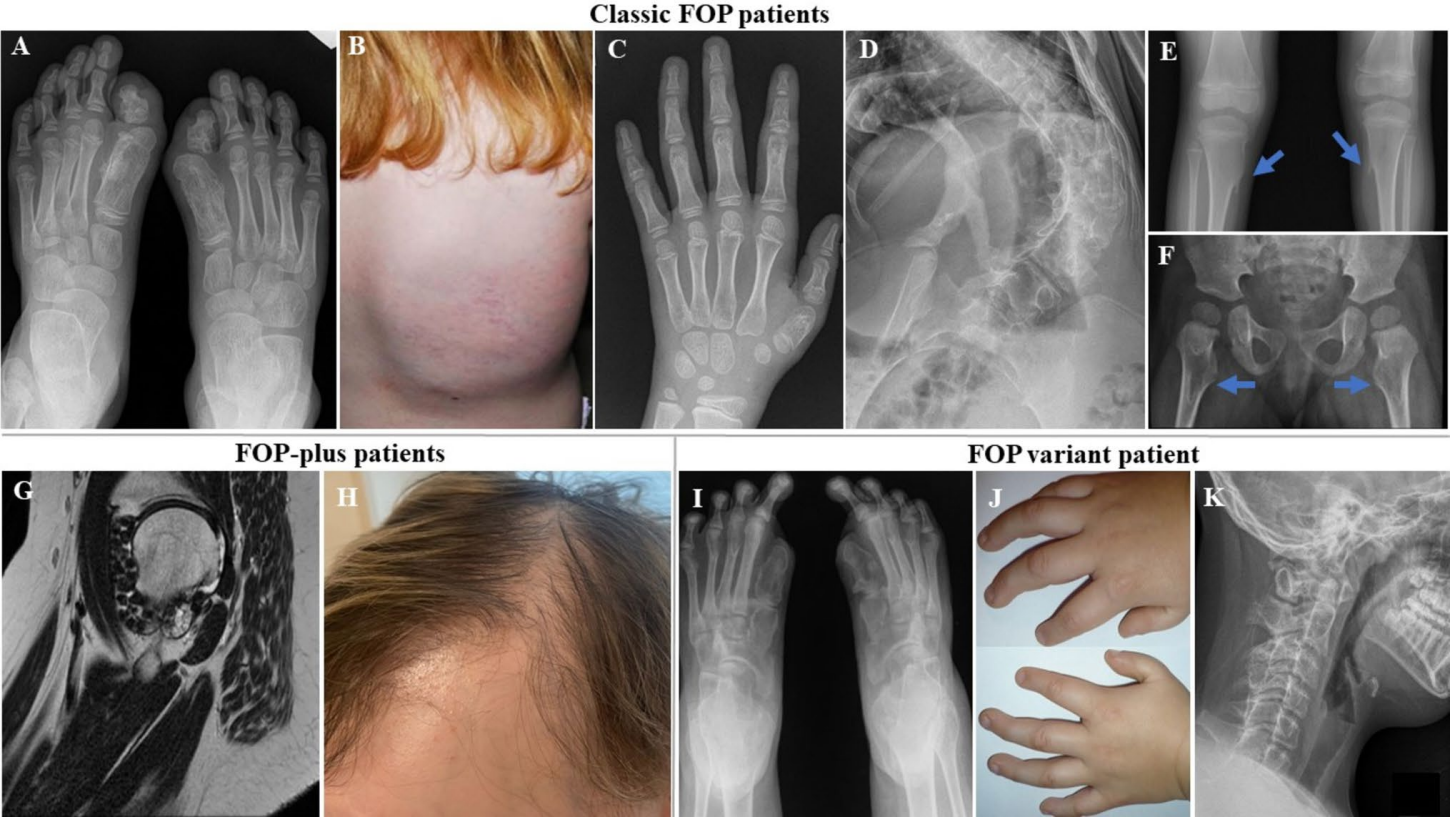
Clinical features of the studied patients. Monophalangism and hallux valgus of both great toes (**A**), heterotopic ossification within the soft tissues of the right scapula (**B**), short first metacarpal bone of the thumb (**C**), severe scoliosis (**D**), proximal medial tibial osteochondromas (**E**), and short broad femoral neck (**F**) in the patients with classical phenotype. Intraarticular synovial chondrosarcoma in the right hip (**G**) in the patient 5 and sparse, slow-growing scalp hair (**H**) in the patient 1. Bilateral absence of both great toes (**I**), shortened thumbs and fifth fingers (**J**), and cervical vertebral malformations (**K**) in the patient 15

Nine patients had the classic FOP phenotype. Seven individuals (P2, P7, P8, P10, P11, P12, and P13) harbored the recurrent pathogenic variant in *ACVR1*, i.e., p.Arg206His, while two patients (P14, and P16) carried unique missense pathogenic variants, i.e., p.Gly356 Asp and p.Arg258Ser. All patients in this cohort exhibited two defining clinical features of FOP, i.e., characteristic malformations of the great toe (Fig. 4A) and HO in specific anatomic patterns (Fig. 4B). They also showed common features of FOP, including thumb malformations (Fig. 4C), spinal malformations (Fig. 4D), tibial osteochondromas (Fig. 4E), and short, broad femoral neck (Fig. 4F). In addition, conductive hearing impairment was present in 78% of the patients.

#### Patients with FOP-plus

Five patients (P1, P3, P4, P5, and P9) had the FOP-plus phenotype. All patients carried the p.Arg206His pathogenic variant in the *ACVR1* gene and displayed classic features of FOP and one atypical feature. Patient 5 was diagnosed with intraarticular synovial chondrosarcoma in her right hip (Fig. 4G), resulting in limited motion. The remaining patients shared common atypical features, i.e., sparse, slow-growing scalp hair (Fig. 4H), which became more pronounced during the second or third decade of life.

#### Patient with FOP variant

Patient 15, with the FOP variant phenotype, harbored the p.Gly356Asp pathogenic variant in *ACVR1*. He presented with bilateral absence of both great toes (Fig. 4I) and shortened thumbs and fifth fingers (Fig. 4J). In addition, ulnarization of the distal phalanges in the third and fourth fingers of both hands (Fig. 4J) was observed. The patient demonstrated limited motion in his cervical spine (Fig. 4K), leading to a forward head posture. He was experiencing difficulty in moving due to contractures in both elbows and knee joints. Furthermore, the individual exhibited facial dysmorphia.

## Discussion

This paper presents the genotype and phenotype of 16 newly reported patients affected by FOP. FOP is an extremely rare condition, with an incidence rate reported at 1 in 2 million. Given this statistic, our cohort of 16 patients accounts for approximately 86% of the expected number of FOP patients in Poland, a country with a population of 37 million. The study encompasses the largest cohort of Polish patients with this condition.

The classical pathogenic variant in *ACVR1* (c.617G>A, p.Arg206His) contributes to most FOP cases (21). We showed that a classical pathogenic variant was present in 81% of our cohort, while two patients (No. 14 and 15) carried a c.1067G>A (p.Gly356Asp) pathogenic variant, and one patient (No. 16) harbored a c.774G>C (p.Arg258Ser) pathogenic variant. All these pathogenic variants have been already reported in the medical literature (Shore et al. 2006; Bocciardi et al. 2009; Furuya et al. 2008). The disorder occurred de novo in all presented cases, and this inheritance pattern was consistent with the previously published cases (Shore et al. 2006). Structural analysis of the ACVR1 protein revealed the mechanisms through which atypical pathogenic variants reported in our patients contribute to FOP. Bocciardi et al. indicated that Arg258 is a surface residue located in the protein kinase domain of ACVR1. The p.Arg258Ser pathogenic variant leads to changes in the stability of the GS region, decreasing the binding affinity of the GS domain with FKBP12, thus causing a gain-of-function effect. Researchers have shown a similar mechanism for the p.Gly356Asp pathogenic variant, which disrupts the stability of the αC helix in the kinase domain of ACVR1 (Bocciardi et al. 2009).

All the presented patients exhibited progressive HO within connective tissues. The average onset age of HO was 3.7 years, aligning with the international average reported at 5 years. All patients presented with a spectrum of clinical FOP symptoms, including progressive HO, malformations of the great toe and thumb, short and broad femoral neck, tibial osteochondromas, and cervical vertebral malformations. Given the above, the most commonly observed clinical feature in our cohort was skeletal phenotype, which corroborates previous findings (Khan et al. 2021). Non-skeletal symptoms of the disease, including neurological and cardiopulmonary dysfunctions, occur less frequently in patients with FOP (Khan et al. 2021). We noted restrictive pulmonary disease in nine patients (P3, P4, P7, P8, P9, P10, P11, P12, and P13), a patent ductus arteriosus in P7, and mitral valve regurgitation in P16. The neurological symptoms that have not been reported in FOP patients include disinhibition in P1 and obsessive–compulsive symptoms in P3 (Khan et al. 2021).

The genotype–phenotype correlation presented herein is consistent with the previously published FOP cases for all analyzed patients except two (P14 and P16). The classical pathogenic variant was reported in all FOP phenotypes (classic FOP, FOP-plus, and FOP variant) (Hüning and Gillessen-Kaesbach 2014). Phenotypic analysis of our patients with the p.Arg206His pathogenic variant (P1-P13) revealed classic or FOP-plus phenotypes. Patient 15 with the p.Gly356Asp pathogenic variant exhibited the FOP variant phenotype. This observation also aligns with prior studies, which identified atypical pathogenic variants in patients with FOP-plus and FOP variant phenotypes (Kaplan et al. 2009; Rafati et al. 2016; Hüning and Gillessen-Kaesbach 2014; Zhang et al. 2013; Hasegawa et al. 2022; Kaplan et al. 2015). The genotype–phenotype correlations of patients 14 and 16 were the most interesting findings. Both individuals carried non-Arg206His pathogenic variants and showed the classic clinical features of the disease. To our knowledge, only three studies have reported patients with atypical pathogenic variants who had the classical FOP phenotype (Bocciardi et al. 2009; Ratbi et al. 2010; Cappato et al. 2021). The phenotypes of two patients from our cohort and the abovementioned cases differ from other reports and indicate a further need to study the phenotypes in patients with atypical pathogenic variants in the *ACVR1* gene. The number of patients with non-Arg206His pathogenic variants is still relatively small, making it challenging to establish a clear genotype–phenotype correlation within this group.

Other research groups have also described the atypical pathogenic variants reported here. Two patients from our cohort (P14 and P15), along with individuals described by Furuya et al. (2008), Kaplan et al. (2009), Stefanova et al. (2012), and Zhang et al. (2013), had the c.1067G>A (p.Gly356Asp) pathogenic variant in the *ACVR1* gene. P14 is the first case with a p.Gly356Asp pathogenic variant that displays a classical phenotype. P15 demonstrated the FOP variant phenotype and shared multiple features common with the case reported by Kaplan et al. (2009) (patient 17), i.e., bilateral absence of both great toes and hypoplastic thumbs with severe shortening of the first metacarpals. P16 and FOP cases reported by Bocciardi et al. (2009), Morales-Piga et al. (2012), Zhang et al. (2013), Eresen Yazıcıoğlu et al. (2013), and Ratbi et al. (2010) harbored the c.774G>C (p.Arg258Ser) pathogenic variant. One case described by Bocciardi et al. (2009) (P17) and the patient reported by Ratbi et al. (2010) presented similar clinical features to our patient, which are typically associated with FOP.

Many clinical trials are underway to establish effective treatments for FOP (Table 2). The disruption of the BMP signaling pathway, a fundamental molecular cascade implicated in the pathogenesis of FOP, is a promising therapeutic target. The scheme of the BMP signaling pathway and the potential therapeutic options currently under investigation are presented in Fig. 2. Palovarotene, under the brand name Sohonos™, is the first medication approved for patients with FOP. The U.S. Food and Drug Administration (FDA) authorized its use in 2023 to reduce new HO in females aged 8 and older and males aged 10 and older. The approval for specific age groups is due to side effects reported during clinical trials, such as alopecia; arthralgia; back pain; skin, lips, and eye dryness; erythema; pain in extremities; pruritus; rash; headache; skin exfoliation; nausea; musculoskeletal pain; myalgia; hypersensitivity; peripheral edema; and fatigue (Anwar and Yokota 2023). Moreover, palovarotene is a teratogen capable of inducing limb anomalies in a fetus and hurts growth plates, hearing, and eyes in pediatric patients. The drug binds to the RARγ receptor, inducing proteasome degradation of Smad1/5/9(8), which results in silencing of the BMP signaling pathway (Shaikh et al. 2023). Another potential target for treating FOP is the ACVR1 receptor inhibition. Saracatinib is a kinase inhibitor and has demonstrated significant promise in preclinical studies by blocking HO. The drug candidate exhibited proper pharmacokinetic and safety parameters and entered a phase II clinical trial in 2020 (Williams et al. 2021). Other selective inhibitors of the ACVR1 receptor under development are zilurgisertib (INCB000928) and fidrisertib (IPN60130). Zilurgisertib inhibits wild-type and pathological ACVR1, impairing BMP signaling in healthy tissues. Fidrisertib demonstrates superiority by selectively inhibiting the mutant ACVR1 receptor and minimally interfering with the wild-type ACVR1 protein. Phase I clinical trial results indicate that fidrisertib has promising pharmacokinetic and pharmacodynamic properties (Meng et al. 2022). The next treatment strategy blocks the activin A. A human anti-activin A monoclonal antibody–garetosmab (REGN2477) inhibited HO in a mouse model with FOP (Hatsell et al. 2015). Results from the phase II LUMINA- 1 clinical trial showed that the drug reduced new HO lesions and flare-ups in adults with FOP. This trial indicated that the optimal advantage of garetosmab therapy may occur early in the disease progression. Garetosmab is presently undergoing phase III OPTIMA clinical trials. Clinicians should monitor the potential side effects of this drug, as activin A influences critical biological processes, including testicular steroidogenesis, spermatogenesis, ovarian follicle growth, and inflammation (Knight et al. 2012; Hedger and Winnall 2012). Finally, rapamycin inhibits the mammalian target of rapamycin (mTOR) signaling pathway, leading to a blockade of the inflammatory and hypoxic pathways that exacerbate the BMP signaling pathway. Hino et al. (2017) demonstrated that mTOR signaling plays a critical role in the early pathophysiology of FOP, while Maekawa et al. (2020) showed that rapamycin suppressed HO in transgenic mice with FOP. Rapamycin proceeded to phase II/III Japanese clinical trial (UMIN000028429) after promising experiments on animals. The clinical trial results have not yet been published. It is worth mentioning that Kaplan et al. showed disease progression in two patients with classic FOP treated with rapamycin (Kaplan et al. 2018). Another inhibitor of the mTOR pathway, BYL719, prevented HO in both cell cultures and mouse models (Valer et al. 2019). This discovery points to BYL719 as a potential candidate for future clinical trials to treat FOP.

**Table 2:** Ongoing clinical trials in FOP (as of July 2024)

NCT/UMIN number	Drug name	Target	Population	Trail phase	Estimated enrollment
NCT05027802	Palovarotene (Sohonos™)	RARγ	Adults and children with FOP	III	61
NCT05394116	Garetosmab (REGN2477)	Activin A	Adults with FOP	III	66
UMIN000028429	Rapamycin (Sirolimus)	mTORC1	Adults and children with FOP	II/III	20
NCT04307953	Saracatinib (AZD0530)	ACVR1	Adults with FOP	II	20
NCT05090891	Zilurgisertib (INCB000928)	ACVR1	Adults and adolescents with FOP	II	60
NCT05039515	Fidrisertib (IPN60130)	ACVR1	Adults and children with FOP	II	98

Recent research has provided insights into potential treatment strategies for FOP, highlighting novel molecular targets and mechanisms. Inhibition of oxidative phosphorylation with IACS- 010759 successfully prevented HO in mice with a pathogenic variant of the *Acvr1* gene (Sun et al. 2024). A related line of research has focused on suppressing primary cilia in human FOP cells and *Acvr1*^Q207D^, Sox2-Cre, and *Acvr1*^R206H^ FOP mouse models. This approach effectively reduced aberrant bone differentiation and extraskeletal ossification by inhibiting BMP and Hedgehog (Hh) signaling pathways (He et al. 2024). In parallel with these findings, human studies have revealed additional perspectives on therapeutic approaches for FOP. A recent case report described a male carrying the p.Arg206His pathogenic variant in the *ACVR1* gene, exhibiting only minimal symptoms of classic FOP. Researchers hypothesized that the limited severity of symptoms in the patient might result from pathogenic variants in the *MMP- 9* gene (c.59 C>T and c.493G> A). Functional studies on FOP mouse models revealed that MMP- 9 inhibition reduced activin A levels and suppressed HO (Lounev et al. 2024). The second report focuses on the application of a therapy aimed at alleviating the symptoms of FOP. The study documented a Polish patient with FOP who experienced an improvement in symptoms following botulinum toxin type A (BTX-A) injections (Dulski and Sławek 2020). The authors suggested that BTX-A therapy may operate through two distinct mechanisms, i.e., blocking an anti-inflammatory response and exerting an analgesic effect. Promising future avenues for FOP treatment in the preclinical stage include gene editing, gene silencing, gene therapy, stem cell therapy, and immunotherapy (Anwar and Yokota 2023). Clustered regularly interspaced palindromic repeats (CRISPR)/Cas9 method can potentially correct pathogenic variants in the *ACVR1* gene, restoring its proper function (Kawamata et al. 2023). The clinical use of CRISPR/Cas-based gene-editing technology faces challenges due to ethical concerns and the risk of unintended harmful genetic alterations. RNA-based strategies may offer an alternative approach by silencing the pathogenic variants in *ACVR1*. Kaplan et al. (2012) and Takahashi et al. (2012) designed allele-specific small interfering RNAs (siRNAs) against p.Arg206His and p.Gly356Asp pathogenic variants. Another research group generated locked nucleic acid gapmers to reduce the expression of the p.Arg206His pathogenic variant in the cell culture model (Maruyama et al. 2022). Additionally, in the study discussed earlier in our review, which indicated primary cilia as a potential therapeutic avenue in the pathogenesis of FOP, researchers employed a siRNA-based drug targeting *Arl3*. The treatment proved effective in reducing HO formation in *Acvr1*^Q207D^ mice (He et al. 2024). The application of RNA-based treatments is still challenging due to difficulties associated with their delivery in vivo. The next potential treatment strategy for FOP involves gene therapy, a method studied by Yang et al. in mouse models of this condition. Researchers administered a viral vector with a corrected version of *ACVR1* to mice. This gene therapy silenced the BMP signaling pathway and prevented HO in an animal model (Yang et al. 2023). The most significant risk factor of gene therapy is an immune response against the viral vectors, highlighting the need for further clinical research and advancements. Another innovative approach utilizes mesenchymal stem cells (MSCs) and induced pluripotent stem cells (iPSCs). Both methods exhibit the potential for regenerating tissues. The study conducted by Kim et al. (2016) demonstrated that introducing a corrected version of the *ACVR1* gene into FOP patient-derived human iPSCs led to the restoration of proper gene expression within the BMP signaling pathway in these cells. Immunotherapy includes monoclonal antibodies (mAbs) and immune checkpoint inhibitors (ICIs) (Shaikh et al. 2023). Although a recent study indicated that anti-ACVR1 monoclonal antibodies promoted HO in FOP mouse models and thus should not be pursued as a treatment strategy for FOP (Aykul et al. 2022), Kan et al. (2019) demonstrated that ICIs prevented HO in animal models, highlighting the potential importance of these drugs in the context of FOP.

In conclusion, we described 16 Polish patients diagnosed with FOP, 13 carrying the p.Arg206His pathogenic variant, two having the p.Gly356 Asp pathogenic variant, and one harboring the p.Arg258Ser pathogenic variant in *ACVR1*. Our results broaden the phenotypic and genotypic landscape of FOP, providing relevant information about genotype–phenotype correlations of the condition. We showed that the classical pathogenic variants in *ACVR1* are associated with either the classical FOP phenotype or the FOP-plus phenotype. We also described two patients with atypical pathogenic variants who presented classical FOP phenotypes. This phenomenon is infrequently observed in previously published cases and requires further studies involving additional patients affected by FOP. Future research should lay the foundation for definite genotype–phenotype correlation within this subset of patients. Currently, therapeutic strategies for FOP are being investigated, targeting the cellular mechanisms and abnormal pathways that trigger aberrant bone tissue formation. Exploring bone formation signaling pathways beyond the BMP pathway, such as the Wnt/β-catenin and Hh pathways, represents a promising direction for understanding the pathogenesis of FOP and identifying novel therapeutic strategies. Early and timely diagnosis enables effective FOP patient management. Conducting molecular tests to identify disease-causing pathogenic variants in *ACVR1* is crucial for confirming or ruling out the condition. This diagnostic approach enables patients and their families to receive appropriate guidance regarding the disease’s nature, progression, and treatment strategies. Altogether, advances in diagnostic methods, deeper insights into the molecular mechanisms associated with the disease, and the development of innovative therapeutic strategies are crucial for improving the management of FOP.

## Supplementary Information

Below is the link to the electronic supplementary material.

Supplementary file1 (DOCX 16 KB)

## Data Availability

Data generated during this study are included in this published article and its supplementary files.

## Code availability

Data generated during this study are included in this published article and its supplementary files.
