# Supplementary material for: Fibrodysplasia ossificans progressiva: genetic and clinical characterization in a cohort of Polish patients and review of potential therapies
Source: J Appl Genet. 2025 Apr 12;67(1):193–203. doi: 10.1007/s13353-025-00966-4 (PMC12819536; doi:10.1007/s13353-025-00966-4)
Supplement: Supplementary file 1 — Supplementary file1 (DOCX 16 KB) [file 13353_2025_966_MOESM1_ESM.docx]

**Supporting information**

| **Primer** | **Sequence 5’ -> 3’(Hg19)** | **Genomic coordinates** |
| --- | --- | --- |
| *ACVR1*_e3F | TGAATGGCAGTTTGAAGGTG | chr2:158655696-158656102 |
| *ACVR1*_e3R | CCATGCTTGGCCCTATATTT |  |
| *ACVR1*_e4F | ACAGGGGGAGAAGCTCATTT | chr2:158636704-158637411 |
| *ACVR1*_e4R | CCAGGGTGACCTTCCTTGTA |  |
| *ACVR1*_e5F | TGTGTGGTCAGGATCAGGAG | chr2:158634329-158634957 |
| *ACVR1*_e5R | CAAATTCCAACCCTCCAAAA |  |
| *ACVR1*_e6F | AAATGTGAGAGGCCATGGA | chr2:158630094-158630852 |
| *ACVR1*_e6R | TCCTTCTTCCAGAGGAGCT |  |
| *ACVR1*_e7F | CCCAAGCTGAGTTTCTCCAG | chr2:158626470-158627159 |
| *ACVR1*_e7R | TACGATATCCCTGGGAGCTG |  |
| *ACVR1*_e8F | CCTCTTAGGGCAATTGGTCA | chr2:158622205-158622842 |
| *ACVR1*_e8R | TGATGGATGGGGAGATGAAT |  |
| *ACVR1*_e9F | AGTGACCCTGGATCCACAAG | chr2:158617156-158617703 |
| *ACVR1*_e9R | AATGGCTGGTCTCTTCCAGA |  |
| *ACVR1*_e10F | CTGCCCTGAGGTATGAGGAA | chr2:158594845-158595214 |
| *ACVR1*_e10R | AGATCCACGGGACAGATCAC |  |
| *ACVR1*_e11F | AGCTTCCAGGGCCATTAAAA | chr2:158593552-158594238 |
| *ACVR1*_e11R | CATTTTGGCAAGTTGGGTCT |  |

**Table S1.** Oligonucleotide primers used in this study
